# Individualized versus standardized risk assessment in patients at high risk for adverse drug reactions (IDrug) – study protocol for a pragmatic randomized controlled trial

**DOI:** 10.1186/s12875-016-0447-6

**Published:** 2016-04-26

**Authors:** Julia Carolin Stingl, Katharina Luise Kaumanns, Katrin Claus, Marie-Louise Lehmann, Kathrin Kastenmüller, Markus Bleckwenn, Gunther Hartmann, Michael Steffens, Dorothee Wirtz, Ann-Kristin Leuchs, Norbert Benda, Florian Meier, Oliver Schöffski, Stefan Holdenrieder, Christoph Coch, Klaus Weckbecker

**Affiliations:** Research Division, Federal Institute for Drugs and Medical Devices, Kurt-Georg-Kiesinger-Allee 3, 53175 Bonn, Germany; Centre for Translational Medicine, University of Bonn, Sigmund-Freud-Str. 25, 53127 Bonn, Germany; Institute of General Practice and Family Medicine, University of Bonn, Sigmund-Freud-Str. 25, 53127 Bonn, Germany; Institute of Clinical Chemistry and Clinical Pharmacology, University of Bonn, Sigmund-Freud-Str. 25, 53127 Bonn, Germany; Department of Economics and Management, Wilhelm Löhe University of Applied Sciences, Merkurstraße 41, 90763 Fürth, Germany; Department of Health Management, University of Erlangen-Nürnberg, Lange Gasse 20, 90403 Nürnberg, Germany

**Keywords:** Individualized medicine, Adverse drug reaction, Elderly, Pharmacogenetics, Polymedication, Drug interaction, ADR risk assessment, Clinical decision support system

## Abstract

**Background:**

Elderly patients are particularly vulnerable to adverse drug reactions, especially if they are affected by additional risk factors such as multimorbidity, polypharmacy, impaired renal function and intake of drugs with high risk potential. Apart from these clinical parameters, drug safety and efficacy can be influenced by pharmacogenetic factors. Evidence-based recommendations concerning drug-gene-combinations have been issued by international consortia and in drug labels. However, clinical benefit of providing information on individual patient factors in a comprehensive risk assessment aiming to reduce the occurrence and severity of adverse drug reactions is not evident. Purpose of this randomized controlled trial is to compare the effect of a concise individual risk information leaflet with standard information on risk factors for side effects.

**Methods/Design:**

The trial was designed as a prospective, two-arm, randomized, controlled, multicenter, pragmatic study. 960 elderly, multimorbid outpatients in general medicine are included if they take at least one high risk and one other long-term drug (polymedication). As high risk “index drugs” oral anticoagulants and antiplatelets were chosen because of their specific, objectively assessable side effects. Following randomization, test group patients receive an individualized risk assessment leaflet evaluating their personal data concerning bleeding- and thromboembolic-risk-scores, potential drug-drug-interactions, age, renal function and pharmacogenetic factors. Control group patients obtain a standardized leaflet only containing general information on these criteria. Follow-up period is 9 months for each patient. Primary endpoint is the occurrence of a thromboembolic/bleeding event or death. Secondary endpoints are other adverse drug reactions, hospital admissions, specialist referrals and medication changes due to adverse drug reactions, the patients’ adherence to medication regimen as well as health related quality of life, mortality and resulting costs.

**Discussion:**

Despite extensive evidence of risk factors for adverse drug reactions, there are few prospective trial data about an individualized risk assessment including pharmacogenetic information to increase patient safety. By conducting a health economic analysis, we will evaluate if the application of an individualized drug therapy in daily routine is cost-effective.

**Trial registration:**

German Clinical Trials Register: DRKS00006256, date of registration 09/01/15.

## Background

Adverse drug reactions (ADR) represent a major public health issue not only due to the morbidity and mortality but also due to the extra costs they cause. About 5–10 % of all hospital admissions are estimated to be ADR-associated, 2–6 % of them are fatal [1–5]. According to analyses of ADR case reports, approximately 40–60 % of all ADR are considered to be preventable [2, 3, 5, 6]. It has been reported that the incidence of ADR increases with age [1, 4–6]. This may be explained by age-related changes in pharmacokinetics, such as increased drug concentrations due to decreased total body water and impaired renal function [7, 8]. Additionally, the prevalence of multimorbidity and polypharmacy is very high in the elderly [9, 10]. In a recent systematic review on ADR in the elderly, these two parameters have been mentioned as significant risk factors for ADR, alongside female sex, impaired renal function, drug-drug-interactions and drugs with narrow therapeutic index, e. g. anticoagulants [11].

Pharmacogenetic factors are known to influence dosing and drug efficacy in patients [12]. For example, clopidogrel effectiveness depends on its transformation into an active metabolite by the genetically variable enzyme cytochrome P 450 2C19 (CYP2C19). Reduced enzyme function has been shown to be significantly associated with decreased platelet inhibition [13–15]. For vitamin K antagonists, cytochrome P 450 2C9 (CYP2C9) is the main drug metabolizing enzyme, and vitamin K epoxide reductase (VKORC1) represents the drugs’ target. They have been shown to significantly influence dosing requirements and anticoagulation stability [16–18]. Evidence-based recommendations for these drugs according to the respective genotypes have been published by the Royal Dutch Pharmacists Association - Pharmacogenetics Working Group [19] and the Clinical Pharmacogenetics Implementation Consortium (CPIC) [20]. The European Medicines Agency (EMA) and the U.S. Food and Drug Administration (FDA) have included pharmacogenetic information for clopidogrel in the drug labels, the FDA also for the vitamin k antagonist warfarin [21, 22].

Another measure to increase patient safety is the use of computerized clinical decision support systems. They have been shown to be a cost-effective possibility to reduce preventable ADR and medication errors in inpatient and ambulatory care [23–26]. Furthermore, some randomized controlled trials investigating the effect of a comprehensive medication review in elderly populations have suggested a reduction of medication-related hospital admissions, but the studies were underpowered and the results were not statistically significant [27, 28]. Therefore, a randomized controlled trial with a large cohort of patients at particularly high risk for ADR is needed for further investigation.

The aim of this study is to test if individualization of drug therapy based on the most important categories of relevant known ADR risk factors may improve safety and individual efficacy of drug therapy if the doctor and the patient are getting this information in a timely and easy-to-understand format. We focus our study on a collective of elderly, multimorbid, polymedicated ADR-high risk patients. As antithrombotics range among the most frequently ADR-associated drugs leading to hospitalizations in the elderly [29, 30] and have high potential for particularly severe ADR [31, 32], these drugs were determined as index medication for the study.

## Methods/Design

### Study aim

The aim of the study is to evaluate the effect of an individualized comprehensive risk assessment and information leaflet regarding medication compared to a standardized one on adverse drug reactions in an elderly high risk population.

### Design

The IDrug-study is a prospective, multicenter, two-arm, randomized, controlled, pragmatic trial. Eligible patients are randomized to either test or control group. Control group patients receive a standardized information leaflet about risk factors for ADR. An individualized leaflet containing the same general information plus an additional individualized risk assessment is handed out to test group patients. In the following passages, the study design will be described in detail.

### Study endpoints

Primary endpoint is the occurrence of a thromboembolic or bleeding event or death within a 9 months study period. Secondary endpoints are the occurrence of other ADR, the number of hospital admissions due to ADR, the number of specialist referrals due to ADR, the number of medication changes, the patients’ adherence to the medication regimen, time to death, health-related quality of life, costs of medication and of additional general practitioner (GP) consultations and hospital treatments as well as a cost-benefit analysis.

### Inclusion and exclusion criteria

Patients are eligible if they are at least 60 years old, have more than one chronic disease and take two or more prescription drugs. They have to be on long-term treatment with oral anticoagulants (phenprocoumon, warfarin, dabigatran, apixaban, rivaroxaban) or antiplatelets (clopidogrel, prasugrel, ticagrelor, ticlopidin) as high risk index medication. Furthermore, they must be physically and mentally able to give written consent to participate in the study. Patients will be excluded if they are unable to give consent or unable to fill in the required questionnaires (SF-36 for health-related quality of life [33], Morisky score for adherence to medication regimen [34] and social state).

### Sample size

Sample size calculation was based on the primary endpoint and its primary analysis. Preceding studies reported incidence rates ranging from 8 to 10.6 % for thromboembolic and bleeding events in a population of elderly polymedicated patients on oral anticoagulants [32, 35]. Thus, for sample size calculation, we assumed an incidence rate of 10 % in the control group, which we assumed to be reduced by half as a result of the individualized risk assessment (i.e. to 5 %). To detect this anticipated difference with a power of 80 % using the Cochran-Mantel-Haenszel test (assuming equal effects in all strata), a sample size of 435 patients per arm is required. Assuming a dropout rate of 10 % about 960 patients need to be included in the study.

### Recruitment

**Fig. 1:**
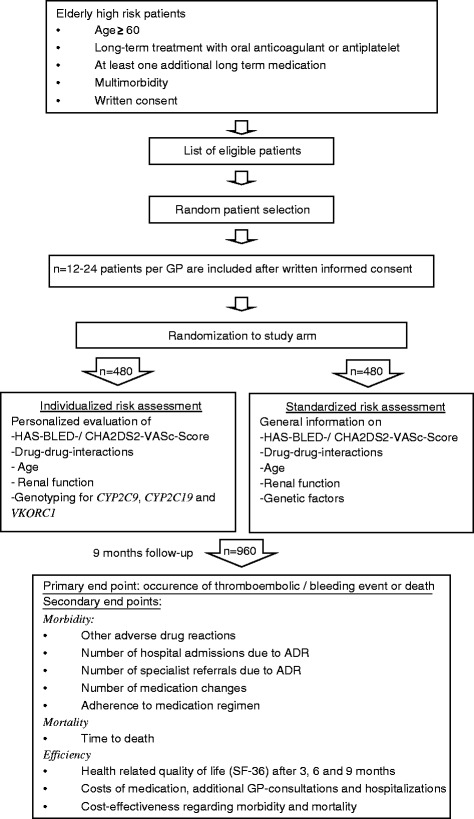
Workflow IDrug study. Each GP provides a list of all patients meeting the inclusion criteria. The order of patient enrollment is random. Following randomization to a study arm, the patients receive either a standardized or an individualized risk assessment leaflet and are followed up for 9 months. 870 patients will be required, and considering an estimated dropout rate of 10 % 960 patients need to be included. At the end of the study, both groups will be compared regarding primary and secondary endpoints

The study is conducted in the area of Bonn, Cologne and the Rhine-Sieg-district, Germany. 40–80 GP-practices situated in this area participate in the study. After being instructed by the Institute of General Practice and Family Medicine Bonn, the general practitioners create a list of eligible patients. They name the number of patients to the institute and in return obtain in a first step a random selection of 10 patients to be asked by the GP to participate in the study. This random selection is performed using prefabricated lists provided by the Department of Biostatistics of the Federal Institute for Drugs and Medical Devices Bonn to avoid selection bias. Overall each GP-practice is planned to enroll 12–24 patients. The workflow of the IDrug study is illustrated in Fig. 1.

### Patient information and inclusion

Written informed consent is obtained by the general practitioner. After enrollment, a blood sample is taken and sent to the accredited Institute of Clinical Chemistry and Clinical Pharmacology of the University of Bonn for genotyping (*CYP2C9, CYP2C19* and *VKORC1*). The patient’s medical history is documented on paper-based Case Report Forms (CRF). The collected data include the patient’s demographic data, such as age, gender, weight, height, blood test results (creatinine, thrombocytes, hemoglobin and liver enzymes), alcohol consumption, smoking state, anticoagulation/antiplatelet therapy regimen, medication and diagnoses. Additionally, the HAS-BLED-Score [36] for bleeding risk (hypertension, abnormal renal/liver function, stroke, bleeding history or predisposition, labile internationalized normalized ratio, elderly (>65 years), drugs/alcohol concomitantly) and the CHA2DS2-VASc-Score [37] for thromboembolic risk (congestive heart failure, hypertension, age, diabetes, stroke, vascular disease, age, sex category) are recorded. Once completely filled in, the CRF is faxed to the Federal Institute for Drugs and Medical Devices Bonn for randomization and risk assessment.

### Randomization

Randomization is performed upon receipt of the CRF and is stratified by GP-practice and gender to account for potential influence of GP and gender and to ensure balanced groups within each stratum. Randomization lists are provided by the Biostatistics Unit of the Federal Institute for Drugs and Medical Devices Bonn. According to the allocated study-arm, either a standardized or an individualized risk assessment leaflet is written and sent out to the GP in duplicate, as one copy will be handed out to the patient.

### Risk assessment

Both risk assessment leaflets contain general information on risk factors for adverse drug reactions. However, the individualized leaflet also includes an additional personalized risk evaluation which takes the patients clinical data into account. For test group patients, their individual HAS-BLED- and CHA2DS2-VASc-Scores are calculated. The current medication is checked for potential drug-drug-interactions and it is checked whether dose adjustments related to the patients’ kidney function would apply, using two independent validated evidence-based clinical decision support systems, AiDKlinik® [38] and the ABDA database [39]. Furthermore, the patients’ genetic profiles for *CYP2C9*, *CYP2C19* and *VKORC1* are matched with their medication to check for potential drug-gene-interactions according to their genotypes. References are the guidelines published by the Royal Dutch Pharmacists Association - Pharmacogenetics Working Group [19] and the Clinical Pharmacogenetics Implementation Consortium (CPIC) [20]. The standardized leaflet, however, only contains general information about these risk factors for adverse drug reactions.

**Fig. 2:**
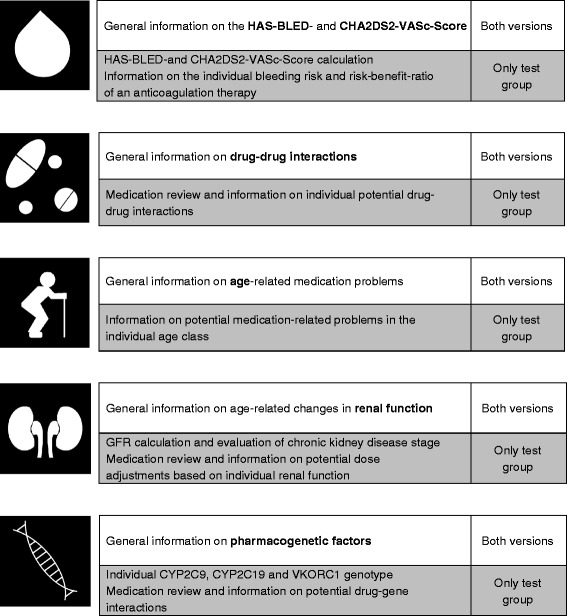
Scheme of the risk assessments. Both versions of risk assessment leaflets contain general information on the following risk factors for ADR: HAS-BLED- and CHA2DS2-VASc-Score, drug-drug-interactions, age, renal function and pharmacogenetic factors. The individualized risk assessments additionally include a personalized evaluation of the patient’s personal data

The leaflets have a short and clearly arranged design in order to be implementable in everyday practice. They only contain clinically relevant information. Additionally, they are written in a generally understandable manner, so that they can be read easily by doctors as well as patients. A scheme of the risk assessment leaflets is displayed in Fig. 2.

### Follow-up and blinding

Follow-up time is 9 months for each patient. Overall, 4 visits are scheduled at intervals of 3 months. The study starts at visit 1 when the patient receives the risk assessment leaflet. During this visit, the general practitioner hands out the leaflet and explains its contents to the patient. It is at the discretion of the GP in which way and to what extent he uses the provided information for further treatment, no specifications are made. Patients are considered to be blinded to group assignment, since group assignment is not actively communicated at any time and both risk assessment leaflets have a very similar layout.

During each visit, the following information is documented in the CRF: reason for GP-consultation, vital signs, ECOG score for the patient’s physical condition [40], adverse events (bleeding events, thromboembolic events and others), number of GP-consultations in the meantime, specialist referrals, hospital admissions, sick leaves, remedies and aids and other procedures (e. g. emergency department visits, rehabilitation etc.). Data about anticoagulation/antiplatelet therapy regimen, medication and diagnoses are updated during each visit. Additionally, patients are asked to fill in questionnaires about their health related quality of life (SF-36 [33], filled in at each visit), their adherence to the medication regimen [34] (filled in at the beginning and end of the study) and their social state (filled in once at the beginning of the study).

### Data management and monitoring

The patients’ data is documented on paper-based Case Report Forms by the general practitioners, the practice staff and staff of the Institute of General Practice and Family Medicine Bonn. It is transferred to a GCP conform electronic database by the data management of the Federal Institute for Drugs and Medical Devices Bonn. To guarantee data quality, double data entry is performed. Queries are documented in an electronic database and processed during monitoring visits. For each practice, three of these visits are scheduled. The first one takes place after inclusion of 1 to 3 patients, the second one after inclusion of 10 to 15 patients and the third one when the practice finishes the study. Furthermore, practices are monitored via telephone and additional interim visits regularly. Monitoring is performed by the Study Center of the Institute of Clinical Chemistry and Clinical Pharmacology, University of Bonn.

### Dropout criteria

Study participation is terminated if the patient withdraws consent or if continuous data collection cannot be guaranteed (e. g. if the patient changes the GP or is absent for a long time). Discontinuation of anticoagulant/antiplatelet therapy after start of the study and receipt of the risk assessment leaflet (visit 1) does not lead to exclusion.

### Ethic approval, data protection and funding

The IDrug study has been approved by the Ethics Comittees of the University of Bonn, of the Medical Association of North Rhine and of the Medical Association of Rhineland-Palatinate. It is performed according to the study protocol, ICH-GCP criteria, EU directives and applicable legal requirements. According to the Declaration of Helsinki, written consent is obtained after oral and written information. Person-identifying data, such as names and birthdays, remain at the GP-practices at all times. All data will be stored for 10 years. Patient information is pseudonymized before transfer to the Federal Institute for Drugs and Medical Devices or the Institute of Clinical Chemistry and Clinical Pharmacology. The IDrug study is financially supported by the Federal Ministry of Education and Research (BMBF). The protocol has been peer-reviewed in a strictly competitive process with several external reviewers from non-German countries evaluating the grant applications.

### Statistical analysis

#### Primary endpoint

The proportion of patients with an event (i. e. thromboembolic or bleeding event or death) will be compared between the two study arms using the Cochran-Mantel-Haenszel-Test stratified by gender and medical practice. Following the intention-to-treat principle, all randomized patients will be analyzed according to their randomized allocation. Dropouts will be counted as treatment failure in primary analysis. Risk reduction is estimated by the Mantel-Haenszel estimator of the common relative risk with the corresponding 95 % confidence interval. Additional sensitivity analyses will be performed, which include evaluation of different covariate structures.

### Secondary endpoints

The SF-36-questionnaire for health related quality of life will be analyzed with a mixed model for repeated measurements using an unstructured covariance matrix . The mixed model contains the categorical covariates treatment, visit, gender and medical practice, a continuous covariate for the baseline SF-36-score and a treatment by visit interaction. Evaluation of drug-associated morbidity involves descriptive analysis of the number of patients with severe adverse drug reactions, the number of hospital admissions and specialist referrals due to ADR and the number of medication changes during the study period. The adherence scale will be evaluated using the non-parametric Wilcoxon-test. Survival time will be analyzed using a log-rank-test stratified by gender and medical practice. Additionally, a Cox-regression, which includes covariates treatment, gender, medical practice, age group and anticoagulant as well as a treatment by gender interaction, will be performed as a sensitivity analysis. The direct costs of the prescribed medication, unexpected GP-visits and hospitalizations will be collected and used for a cost-effectiveness analysis to compare the two study groups. In this context, two types of health outcomes will be examined. Firstly, the incidence of adverse events and secondly, the quality-adjusted-life-years (QALY) measures using the SF-36-questionnaire.

## Discussion

Objective of the IDrug study is to further investigate individual patient ADR risk factors in a randomized controlled fashion in a real-world clinical setting with elderly patients at high risk for ADR. In contrast to preceding clinical studies, clinical and pharmacogenetic risk factors are not considered in isolation, but together resulting in a comprehensive medication review in form of a risk assessment leaflet. The pragmatic study design does not only allow to obtain valuable data with high external validity, but also paves the way for clinical applicability. For the purpose of realistic conditions, GP-practices were chosen as study sites. Due to the inclusion criteria a broad representative patient population can be acquired. The high risk characteristic of the study cohort might enhance detection rates of adverse drug reactions.

One limitation of the study is that the degree of accuracy of blinding might decrease over the study period, as each GP will receive both versions of risk assessment leaflets and, therefore, might be able to identify study groups during the course of the trial. In contrast, patients only get to see their own risk assessment leaflets. Therefore, it is less likely that blinding is broken for patients. Furthermore, although the provided risk assessment covers the most important risk factors for ADR, some parameters are not included, e. g. drug-disease interactions and missing drug indications. The reason is to keep the leaflets as short as possible to ensure the GP’s and patient’s motivation to read it.

To our knowledge, IDrug is the first randomized controlled trial evaluating the impact of a comprehensive individualized risk assessment including genotyping on adverse drug reactions in a large elderly high risk population. Based on the study results, we will evaluate whether this individualized approach has the potential to increase patient safety in daily routine by reducing ADR in a cost-effective way. This may advance development of rational, evidence-based policies for the application of pharmacogenetic testing in individualized drug therapy in clinical practice.

## Abbreviations

ADR: Adverse drug reaction(s)
CHA2DS2-VASc: Congestive heart failure, hypertension, age, diabetes, stroke, vascular disease, age, sex category
CRF: Case report form(s)
CYP: Cytochrome P450 enzyme
DNA: Deoxyribonucleic acid
ECOG: Eastern cooperative oncology group
EMA: European Medicines Agency
EU: European Union
FDA: U.S. Food and Drug Administration
GCP: Good clinical practice
GP: General practitioner
HAS-BLED: Hypertension, abnormal renal/liver function, stroke, bleeding history or predisposition, labile internationalized normalized ratio, elderly (>65 years), drugs/alcohol concomitantly
ICH: International conference of harmonization
INR: International normalized ratio
QALY: Quality-adjusted-life-years
SF: Short form
VKORC: Vitamin K epoxide reductase complex
